# Societal costs of non-cardiac chest pain compared with ischemic heart disease - a longitudinal study

**DOI:** 10.1186/1472-6963-13-403

**Published:** 2013-10-09

**Authors:** Ghassan Mourad, Jenny Alwin, Anna Strömberg, Tiny Jaarsma

**Affiliations:** 1 Department of Social and Welfare Studies, Division of Health, Activity and Care, Faculty of Health Sciences, Linköping University, Linköping, Kungsgatan 40, 601 74, Norrköping, Sweden; 2 Department of Medical and Health Sciences, Division of Healthcare Analysis, Faculty of Health Sciences, Linköping University, Linköping, Sweden; 3 Department of Medical and Health Sciences, Division of Nursing Science, Faculty of Health Sciences, Linköping University, Linköping, Sweden

**Keywords:** Non-cardiac chest pain, Ischemic heart disease, Healthcare utilization, Societal costs, Direct costs, Indirect costs

## Abstract

**Background:**

Non-cardiac chest pain (NCCP) is a common complaint. Our aim was to present a detailed description of the costs of patients with NCCP compared to patients with acute myocardial infarction (AMI) and Angina Pectoris (AP) from a societal perspective.

**Methods:**

Data on healthcare utilization and annual societal costs, including direct healthcare costs and indirect costs due to productivity loss, were collected from different databases. The participants consisted of 199 patients from a general hospital in Sweden (99 with NCCP, 51 with AMI, 49 with AP), mean age of 67 years, 59% men.

**Results:**

NCCP, AMI, and AP patients had on average 54, 50 and 65 primary care contacts and 3, 4, and 4 hospital admissions during a period of 2 years. Length of hospital stay was 6, 11 and 11 days. On average, 14%, 18%, and 25% of NCCP, AMI and AP patients were on sick-leave annually, and about 12% in each group received a disability pension. The mean annual societal costs of NCCP, AMI and AP patients were €10,068, €15,989 and €14,737.

**Conclusions:**

Although the annual societal cost of NCCP patients was lower than in AMI and AP patients, the cost was still considerable (€10,068). Taken into account the high prevalence of NCCP, the cumulative annual national cost of these patients could be more than the double of AMI and AP if all patients incurred the same costs as in this study. Targeted interventions are important in order to support patients with NCCP and minimize healthcare utilization and costs.

## Background

Non-cardiac chest pain (NCCP) can be defined as angina-like pain that has not been diagnosed as ischemic heart disease [1]. It can be caused by gastrointestinal diseases, pulmonary disorders, chest wall syndromes, and pleural and pericardial conditions. NCCP is often also associated with psychiatric disorders and has been found to decrease quality of life [2-4].

Patients suffering from NCCP are often younger, female, immigrants, and have a lower educational level compared to patients suffering from ischemic heart disease [1,5,6]. The prevalence rate of NCCP is estimated to be more than 50% of all chest pain cases presenting at the emergency department [3,7,8], which in turn is between 2% and 5% of all cases [9].

Patients with NCCP often seek care due to recurrent and persistent chest pain [10-15]. Other reasons for seeking care reported by patients are symptom anxiety, anxiety due to the possibility of a serious disease and symptom severity [1]. Patients suffering from NCCP have been found to utilize out-patient healthcare to the same extent as patients with acute myocardial infarction (AMI) [2,4,15]. The majority of the patients seeking care due to chest pain are admitted for in-hospital cardiac “rule out” observation, but only one third of all cases are found to be ischemic heart disease [12,16]. In many cases, patients remain undiagnosed despite investigation [17], even though they could suffer from depressive symptoms [15].

A minority of NCCP patients are high users of healthcare [2,5,7,15] and contribute to high costs for the healthcare system [9,16,18]. According to Glombiewski et al. [12], these patients have an inappropriate healthcare usage, defined as 2 or more visits to a cardiologist, or 3 or more cardiac diagnostic investigations within a period of 6 months. Yet, there is no research on the societal costs related to patients with NCCP, including costs of the patients’ healthcare utilization and productivity loss. By calculating the societal costs of NCCP, valuable information is obtained that can play a central role in deciding the urgency to develop new treatments for these patients in order to optimize the patient benefits from the money spent. The aim of the present study was to present a detailed description of the costs of patients with NCCP compared to patients with AMI and Angina Pectoris (AP) from a societal perspective.

### Research questions

(1) How much of healthcare resources do patients with NCCP, AMI and AP utilize? What are the differences between the index admission year and the following year?

(2) What are the direct and indirect costs of patients with NCCP, AMI and AP? What are the differences between the index admission year and the following year?

(3) What are the annual societal costs for patients with NCCP, AMI and AP?

(4) Which are the most cost driving units within primary and hospital care in connection with patients with NCCP, AMI and AP?

## Methods

A longitudinal descriptive study of the costs of patients with NCCP, AMI, and AP over a 2-year period was performed. A societal perspective, including all costs of the patients’ healthcare utilization and productivity loss, was applied in the analysis.

### Enrolment

**Figure 1:**
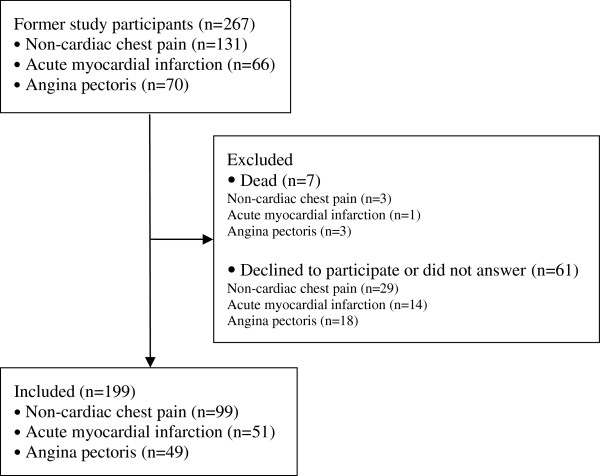
Flow diagram illustrating patient enrolment.

Two hundred and sixty seven patients who had been admitted to a medical unit, mainly the coronary care unit at a general hospital, due to acute chest pain between July and December 2008 and discharged with ICD- diagnoses (International Classification of Diseases) of NCCP (ICD 10-code R07.4, chest pain unspecified; and ICD 10-code Z03.4, observation for suspected myocardial infarction), AMI (ICD 10-code I21) and AP (ICD 10-code I20), were approached (Figure 1). The cohort was prevalence-based, meaning that all patients diagnosed with NCCP, AMI or AP during the study period were approached [19]. These patients had participated in a previous study regarding depressive symptom prevalence and healthcare utilization [15]. At the 1-year follow-up, 260 surviving patients of the 267 who had been approached were invited to participate in the present study. All participants were residents of the County of Östergötland, which is the fourth largest county in Sweden with 429,642 residents. About 19% of Östergötland residents are older than 65, mean age 41 years, and the employment rate is 74% [20].

### Data collection

#### Cost estimates

When estimating cost-of-illness, the cost-generating components are identified and attributed a monetary value. When assessing the total economic costs of illness, these components are the direct costs and the indirect costs or productivity losses. The direct costs are related to health care expenditures and the productivity costs measure production lost due to morbidity and mortality [19,21].

All costs are reported in Euros (€) (€1 equals 8.6 Swedish kronor as per January 3, 2013).

#### Direct costs

The direct healthcare costs of patients with NCCP, AMI and AP were defined as all costs related to diagnosis procedures and/or treatment within primary care, out-patient clinics, and hospital care. All healthcare utilization of the patients in the present study was included in the analyses, regardless of the reason for the healthcare contact. The costs are presented as hospital care including nursing/caring staff and premises costs (hospital care), non-nursing staff and treatment (hospital care), staff and treatment (primary and out-patient care), medical service, drugs, materials and medical devices, administrative contacts, and various overhead costs (hospital, primary and out-patient care). Medical service includes anaesthesia, surgery, postoperative care, intensive care, radiology, and laboratory services.

Data on the direct costs were collected from the Cost Per Patient (CPP) database. This regional database, which is run by Östergötland County Council, was introduced by the Swedish Association of Local Authorities and Regions, and provides patient-related cost information about almost all healthcare within the county of Östergötland [22].

The costs provided by the CPP database are based on information from a regional care database called “Care Data Warehouse”. This is a population-based diagnosis-related administrative database run by Östergötland County Council. The medical diagnoses are recorded according to ICD-10. The Care Data Warehouse database contains information about every healthcare visit/stay within the county of Östergötland, except for some private practices [23,24].

#### Indirect costs

The indirect costs were mainly based on productivity loss due to sick-leave, and also productivity loss due to disability pension in cases of prolonged reduced work capacity. The Human Capital approach, which is the most used method [25], was used when estimating the indirect costs. This means that the estimates were based on gross earnings including employment overheads and benefits of the individuals in employment, and in some cases also of those not in paid employment, e.g. individuals who were unemployed or on parental leave. By using this method, adjustments were made for the opportunity cost of the production the individuals could have contributed with if they had been at work [19,21,26].

Data on the indirect costs were collected from the Social Insurance Agency database. All persons receiving sickness benefit, regardless of employment status, were included in the analysis. It was assumed that these individuals would be back in production. Data regarding sick-leave are registered and paid by the Social Insurance Agency to employed individuals on sick-leave from the 15^th^ day of absence, and from the second day of sick-leave to those who are unemployed or on parental leave. We added the unregistered days to our calculations and the total cost for sick-leave from day 1 is presented in those with a sick-leave period longer than 14 days. Disability pension was paid to people between 30 and 64 years with at least 25% reduced work capacity for at least 1 year [27].

The calculations of productivity loss were based on the patients’ income. These data were obtained from a questionnaire completed by the patients. In the questionnaire, the patients stated their income by marking 1 of 4 pre-defined income ranges (Table 1). The mean value of the income ranges “low, middle-range and high” (€1686, €2500, and €4279) was used in the calculations. For the income range “very low”, the maximum income of €1337 was used in the calculations. An employer’s tax of 50% was added to the income.

**Table 1:** Socio-demographic data of the patients with non-cardiac chest pain, acute myocardial infarction and angina pectoris

	**Socio-demographic data**				
	**All patients**	**Non-cardiac chest pain**	**Acute myocardial infarction**	**Angina Pectoris**	**P-value**
	**(N = 199)**	**(n = 99)**	**(n = 51)**	**(n = 49)**	
Age year (m ± SD)	67 ± 13	64 ± 14	69 ± 12	69 ± 12	.01
Male sex n (%)	117 (59)	48 (49)	35 (69)	34 (69)	.01
Married/cohabiting n (%)	144 (72)	72 (73)	40 (78)	32 (65)	.34
Educational level n (%)					.76
Compulsory school	82 (41)	43 (43)	17 (33)	22 (45)	
High school	78 (39)	38 (39)	22 (43)	18 (37)	
University	39 (20)	18 (18)	12 (24)	9 (18)	
Work status n (%)					.14
Workers	55 (28)	28 (28)	10 (20)	16 (33)	
Retired	120 (60)	55 (56)	34 (66)	31 (63)	
Sick-leave/disability pension	14 (7)	7 (7)	6 (12)	2 (4)	
Unemployed	8 (4)	7 (7)	1 (2)	0 (0)	
Students	2 (1)	2 (2)	0 (0)	0 (0)	
Income n (%)					.92
Very low (≤ €1337)	70 (35)	38 (38)	20 (39)	12 (25)	
Low (€1337-2035)	62 (31)	27 (27)	14 (28)	21 (43)	
Middle-range (€ 2035–2965)	44 (22)	24 (24)	10 (19)	10 (20)	
High (≥ €2965)	23 (12)	10 (10)	7 (14)	6 (12)	

Costs related to an individual due to pain and suffering or to family members for caring for the individual or home care services were not included due to lack of data.

### Procedure

A letter including information about the study, written informed consent and a pre-stamped envelope was sent to all patients (n = 260). They were asked to permit that data were collected from the different databases. After 3 and 6 weeks, a reminder was sent to those who had not responded to the first mail-out. Patients declining participation were not contacted further. Two-year data were collected from the different databases based on the personal identification numbers of the patients who agreed to participate. Data were provided to the researchers on an individual basis and for each contact with healthcare. The data were calculated group-wise and year-wise by the researchers. The 2-year period comprised 1 year including index admission (i.e. admission when patients were recruited) and the year following index admission.

### Statistical analysis

IBM SPSS Statistics 20 was used to perform the statistical analysis. Number, percent, mean and standard deviation (SD) were used to describe the socio-demographic variables. Between-group differences regarding socio-demographic variables were calculated using analysis of variance with the Bonferroni post hoc correction for continuous variables and χ^2^ test for categorical variables. Since there were differences in demographic data, aetiology and treatment needs between the 3 groups, no statistical differences between groups were calculated regarding healthcare utilization and costs, but comparisons were made on a descriptive level in order to highlight and discuss similarities and differences.

Mean values and standard deviations were used to describe healthcare utilization, such as number of primary care and out-patient contacts, hospital admissions and length of hospital stay, as well as direct and indirect costs. Wilcoxon signed rank test was used to analyze differences within groups regarding healthcare utilization and costs over the 2 years. A rough estimate of the index admission costs was made by dividing the total hospital cost for the first year with the number of admissions. A difference of p < .05 was considered significant.

### Ethical considerations

The study was approved by the Regional Ethical Review Board in Linköping, Sweden (code M12-08 and M12-08 T118-09), and was carried out in accordance with the Declaration of Helsinki. Study participation was based on written informed consent, which was obtained from all participants.

## Results

### Study participants

Out of the 260 patients who were approached, a total of 199 (77%; 99 with NCCP, 51 with AMI and 49 with AP) agreed to participate in the present study (Figure 1). The participants ranged in age from 21 to 90 years with a mean age of 67 years. Participants were mainly men (59%), married (72%), retired (61%) and had a low income (66%) (Table 1). Patients with NCCP were significantly younger and did not differ regarding sex compared with the other 2 groups (p < .05). No other differences were found regarding the socio-demographic variables. Non-participants (23%; 29 with NCCP, 14 with AMI and 18 with AP) did not differ from the participants regarding sex, but patients with NCCP were significantly younger (p = .05), and those with AP were significantly older (p = .02).

### Healthcare utilization in patients with NCCP, AMI and AP

During the index admission year, patients with NCCP, AMI, and AP had a mean of about 24, 16 and 27 primary care and out-patient contacts per patient (Table 2). The mean number of primary care and out-patient contacts were about 30, 34 and 38 per patient respectively, during the following year, which was significantly higher (p < .01) than the first year for each group. The causes for these contacts varied and were not only related to the NCCP, AMI and AP diagnoses.

**Table 2:** Healthcare utilization in patients with non-cardiac chest pain, acute myocardial infarction and angina pectoris

	**Non-cardiac chest pain (n = 99)**		**Acute myocardial infarction (n = 51)**		**Angina Pectoris (n = 49)**	
	**Year 1**	**Year 2**	**Year 1**	**Year 2**	**Year 1**	**Year 2**
Mean number of primary care and out-patient contacts per patient (m ± SD)	23.6 ± 27.0	30.0 ± 36.8	16.2 ± 13.4	33.6 ± 23.3	26.5 ± 23.0	38.3 ± 36.1
Mean number of hospital admissions per patient (m ± SD)	1.7 ± 1.4	0.9 ± 1.8	3.0 ± 1.6	0.6 ± 0.9	2.6 ± 1.7	1.3 ± 1.8
Mean length of hospital stay, days per patient (m ± SD)	3.4 ± 5.2	2.9 ± 9.4	9.0 ± 8.8	1.9 ± 3.8	6.8 ± 6.1	3.7 ± 8.3

The first year, patients with NCCP had almost half the number of hospital admissions per patient compared to patients with AMI and AP, who had about 3 admissions per patient. All 3 groups had significantly fewer admissions (P < .001) during the second year compared to the first (Table 2), and also significantly shorter hospital stays the second year compared to the first (p < .01). The causes for the admissions were directly related to the NCCP, AMI and AP diagnoses in 80-85% of the admissions in all groups.

### Direct and indirect costs of patients with NCCP, AMI and AP

All costs within primary care, out-patient clinics and hospital care are specified year-wise in Tables 3, 4 and 5 for each diagnosis group.

**Table 3:** Annual direct cost per patient of non-cardiac chest pain in Euros (€)

	**Cost per patient of non-cardiac chest pain (n = 99)**					
	**Primary care and out-patient clinics**		**Hospital care**		**Total annual direct costs**	
	**Cost per patient**	**Cost per patient**	**Cost per patient**	**Cost per patient**	**Cost per patient**	**Cost per patient**
	**Year 1**	**Year 2**	**Year 1**	**Year 2**	**Year 1**	**Year 2**
Hospital care including nursing/caring staff and premises costs^*^	0	0	1203	1117	1203	1117
Staff and treatment^□^	1356	1530	572	418	1928	1948
Medical service^‡^	477	591	1280	850	1757	1441
Drugs, materials and medical devices	955	1002	408	308	1363	1310
Administrative contacts^†^	104	141	2	8	106	149
Overhead costs and other	321	351	339	262	660	613
**Total cost**	**3212**	**3616**	**3803**	**2963**	**7016**	**6578**

* = Not relevant for primary care and out-patient clinics.

^□^ = In hospital care, the costs for nursing/caring staff are not included.

‡ = Medical service includes anaesthesia, surgery, postoperative care, intensive care, radiology, and laboratory services.

† = Administrative contacts mainly within primary care and out-patient clinics.

**Table 4:** Annual direct cost per patient of acute myocardial infarction in Euros (€)

	**Cost per patient of acute myocardial infarction (n = 51)**					
	**Primary care and out-patient clinics**		**Hospital care**		**Total annual direct costs**	
	**Cost per patient,**	**Cost per patient,**	**Cost per patient,**	**Cost per patient,**	**Cost per patient,**	**Cost per patient,**
	**Year 1**	**Year 2**	**Year 1**	**Year 2**	**Year 1**	**Year 2**
Hospital care including nursing/caring staff and premises costs^*^	0	0	3685	671	3685	671
Staff and treatment^□^	865	1324	2237	343	3101	1667
Medical service^‡^	287	483	8131	1610	8418	2092
Drugs, materials and medical devices	603	912	780	157	1383	1069
Administrative contacts^†^	66	82	6	0	72	82
Overhead costs and other	203	311	1548	322	1751	634
**Total cost**	**2024**	**3112**	**16,387**	**3103**	**18,411**	**6215**

* = Not relevant for primary care and out-patient clinics.

^□^ = In hospital care, the costs for nursing/caring staff are not included.

‡ = Medical service includes anaesthesia, surgery, postoperative care, intensive care, radiology, and laboratory services.

† = Administrative contacts mainly within primary care and out-patient clinics.

**Table 5:** Annual direct cost per patient of angina pectoris in Euros (€)

	**Cost per patient of angina pectoris (n = 49)**					
	**Primary care and out-patient clinics**		**Hospital care**		**Total annual direct costs**	
	**Cost per patient,**	**Cost per patient,**	**Cost per patient,**	**Cost per patient,**	**Cost per patient,**	**Cost per patient,**
	**Year 1**	**Year 2**	**Year 1**	**Year 2**	**Year 1**	**Year 2**
Hospital care including nursing/caring staff and premises costs^*^	0	0	2722	1454	2722	1454
Staff and treatment^□^	1342	1651	1576	545	2918	2196
Medical service^‡^	455	447	4609	1003	5064	1450
Drugs, materials and medical devices	869	784	890	683	1760	1467
Administrative contacts^†^	109	119	4	0	113	120
Overhead costs and other	314	346	984	400	1297	746
**Total cost**	**3089**	**3348**	**10,785**	**4084**	**13,875**	**7432**

* = Not relevant for primary care and out-patient clinics.

^□^ = In hospital care, the costs for nursing/caring staff are not included.

‡ = Medical service includes anaesthesia, surgery, postoperative care, intensive care, radiology, and laboratory services.

† = Administrative contacts mainly within primary care and out-patient clinics.

### Direct costs

#### Primary care/out-patient care

The annual costs for out-patient care at primary care and out-patient clinics were higher the second year compared to the first for patients with NCCP (p < .05) and AMI (p < .01). The mean total cost per patient the first year was €3212 for patients with NCCP, €2024 for patients with AMI and €3089 for patients with AP (Table 6). The corresponding costs for the second year were €3616, €3112, and €3348 for patients with NCCP, AMI and AP respectively.

**Table 6:** Total annual societal cost per patient, in Euros (€)

		**Non-cardiac chest pain (n = 99)**	**Acute myocardial infarction (n = 51)**	**Angina pectoris (n = 49)**
Direct cost: Primary care/out-patient clinics	Total costs per patient year 1	€3212	€2024	€3089
	Total costs per patient year 2	€3616	€3112	€3348
Direct cost: Hospital care	Total costs per patient year 1	€3803	€16,387	€10,785
	Total costs per patient year 2	€2963	€3103	€4084
Indirect cost	Total costs per patient year 1	€3527	€3670	€3652
	Total costs per patient year 2	€3014	€3682	€4516
**Total annual societal cost per patient**		**€10,068**	**€15,989**	**€14,737**

#### Hospital care

During the first year, the mean hospital costs were €3803, €16,387, and €10,785 per patient for patients with NCCP, AMI and AP respectively. The corresponding costs for the second year were €2962, €3103, and €4084. The annual costs for hospital care were lower during the second year compared to the first in patients with AMI and AP (p < .01), but did not differ in patients with NCCP. A rough estimate of the costs of the index admission resulted in sums of €2237, €5462, and €4148 per patient for patients with NCCP, AMI and AP respectively.

### Indirect costs

#### Sick-leave

In total, 21% of the study participants were on sick-leave during the first year and 16% during the second year. In terms of the diagnosis groups, patients with NCCP were on sick-leave 16% and 11% the first and second year respectively (Table 7). The corresponding numbers for patients with AMI were 20% and 16%, and for patients with AP 27% and 22%. On average, patients with NCCP, AMI and AP were on sick-leave 103, 54 and 94 days per year respectively. Although fewer patients were on sick-leave the second year compared to the first, these patients had longer periods of absence. In patients with NCCP, the mean cost per patient due to productivity loss because of sick-leave was €1182 the first year and €1283 the second year, €835 and €972 in patients with AMI, and €1175 and €2803 in patients with AP. No significant differences were found between year 1 and year 2 in any of the groups.

**Table 7:** Productivity loss due to sick-leave and disability pension, in Euros (€)

	**Productivity Loss**					
	**Non-cardiac chest pain (n = 99)**		**Acute myocardial infarction (n = 51)**		**Angina Pectoris (n = 49)**	
	**Year 1**	**Year 2**	**Year 1**	**Year 2**	**Year 1**	**Year 2**
Patients with sick-leave n (%)	16 (16)	11 (11)	10 (20)	8 (16)	13 (27)	11 (22)
Length of sick-leave in days per patient (m ± SD)	86 ± 93	120 ± 123	34 ± 12	73 ± 42	45 ± 33	142 ± 140
Cost of sick-leave in Euros (€) per patient with sick-leave (m ± SD)^*^	7311 ± 6026	11,549 ± 11,913	4256 ± 2006	6195 ± 4744	4429 ± 2416	12,486 ± 13,261
Total productivity loss cost in Euros (€) due to sick-leave	116,976	127,039	42,560	49,560	57,577	137,346
**Cost of sick-leave in Euros (€) per patient in total**	1182	1283	835	972	1175	2803
Patients with disability pension due to illness (%)	10 (10)	9 (9)	6 (12)	6 (12)	7 (14)	6 (12)
Length of disability pension in days per patient (m ± SD)	354 ± 35	322 ± 102	365 ± 0	331 ± 83	322 ± 98	268 ± 154
Cost of disability pension in Euros (€) per disabled patient (m ± SD)^*^	23,220 ± 12,919	19,036 ± 12,116	24,094 ± 7098	23,036 ± 9449	17,342 ± 8291	13,991 ± 11,547
Total productivity loss cost in Euros (€) due to disability pension	232,200	171,324	144,564	138,216	121,394	83,946
**Cost of disability pension in Euros (€) per patient in total**	2345	1731	2835	2710	2477	1713
**Cost of productivity loss in Euros (€) per patient in total**	**3527**	**3014**	**3670**	**3682**	**3652**	**4516**

^*^ = The mean value of the income ranges “low, middle-range and high” (€1686, €2500, and €4279) was used in the calculations. For the income range “very low”, the maximum income of €1337 was used in the calculations.

#### Disability pension

On average, 12% of the study participants drew a disability pension the first year and 11% the second year. There were no substantial differences between the groups (Table 7). On average, patients with NCCP, AMI and AP received a disability pension for 338, 348 and 295 days per year respectively. The mean cost per patient due to productivity loss because of disability in patients with NCCP was €2345 the first year and €1731 the second year, €2835 and €2710 in patients with AMI, and €2477 and €1713 in patients with AP. There were no significant differences between year 1 and year 2 in any of the groups.

### Total annual societal costs of patients with NCCP, AMI and AP

The total annual societal cost per patient, including both direct and indirect costs, is reported in Table 6. The total cost of patients with NCCP was about €10,068 per patient annually. Patients with AMI incurred a cost of €15,989 and those with AP €14,737.

### Cost driving units within primary and hospital care

**Figure 2:**
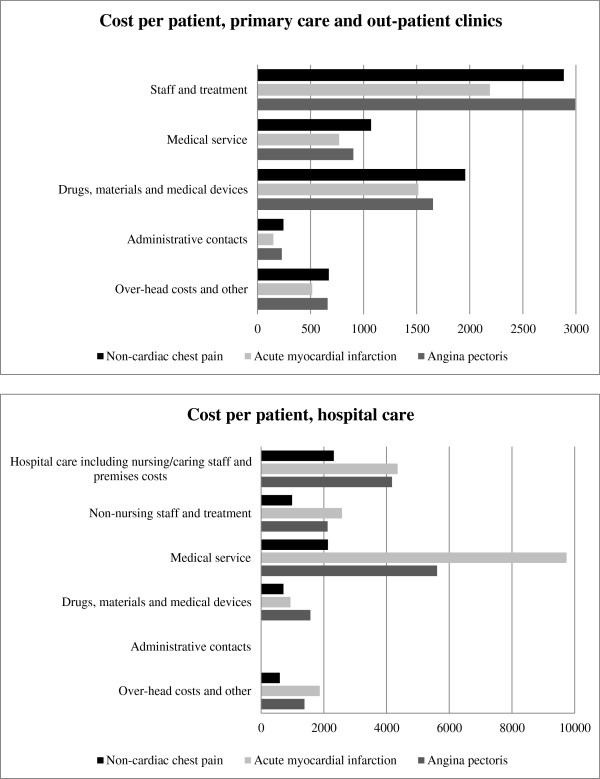
**Cost drivers within primary care and out-patient clinics, and hospital care.** 2-year costs in Euros (€).

The most cost driving units within primary care and out-patient clinics were the physician appointments, which accounted for the main bulk of the staff costs (44%), and the costs for drugs (28%) (Figure 2). The corresponding cost driving units within hospital care were hospital care, including nursing/caring staff and premises costs (26%), and radiology costs, which is part of the medical service (43%).

## Discussion

This study provides a unique and detailed description of the costs associated with patients with NCCP, AMI and AP from a societal perspective. This has not previously been reported. In the present study, we have calculated the annual societal costs, including direct healthcare costs and indirect costs, due to productivity loss associated with NCCP, compared to costs of AMI and AP. Although the majority of patients with NCCP have no cardiac conditions, many of them believe that they do and they are often treated in cardiac units. Therefore, it is of great interest to compare them with cardiac patients. By comparing them with the more severe cardiac patients who require more and expensive healthcare resources, we can show the extent of the costs incurred by those with NCCP, who have a significantly less severe condition. By highlighting the extent of the costs, we can also emphasize the importance of early diagnostics and treatment for these patients which can have a positive impact on healthcare utilization and costs. This can also lead to better use of resources for those with cardiac conditions.

Patients with NCCP incur large costs for the society, that are similar to or exceed those of AMI and AP, particularly with regard to primary care costs and indirect costs due to sick-leave. On average, patients with NCCP had 54 primary care and out-patient contacts during the study period, incurring higher costs than patients with AMI and AP. This is noteworthy since many of these patients do not have a clear cause and medical diagnosis explaining the pain. The lack of explanation for the chest pain can lead to psychological distress, which may explain these patients’ high number of primary care contacts. According to Eslick [2], there is a relationship between seeking behavior, symptom severity and higher level of psychological distress.

All 3 groups had significantly more contacts with primary care/out-patient clinics and incurred higher costs in year 2 compared to year 1. This is expected since most treatment and follow-ups after the index admission are carried out within primary care and out-patient clinics. Another reason is that in Sweden, primary care has a strict gatekeeper role and people are advised to initially consult primary care for most symptoms [28]. However, when they experience chest pain, they can seek acute hospital care without referral from primary care.

Patients with NCCP had fewer admissions and shorter length of hospital stay, and thereby lower hospital costs, than those with AMI and AP, which is similar to earlier research [5,18]. The costs for a hospital admission in the present study was €2602 for 2.4 days for patients with NCCP, to be compared with €884 for about 1 day of hospital admission in a previous study from Sweden [16], and €3729 for 3.8 days in Ireland [18]. All 3 groups had significantly fewer admissions, and in AMI and AP the length of hospital stays were also shorter year 2 compared to year 1, leading to lower costs in all patient groups. This can partly be explained by the fact that the index admission was included in the first year. However, in patients with AMI and AP, the number of admissions and the length of hospital stay may have decreased after patients had received their diagnoses and treatment for these. Although the mean number of admissions and hospital days was low in all patients, it is recommended that strategies are implemented in order to reduce the number of admissions and hospital days since they represent the greatest part of the cost [16,18]. In this study, one of the most cost driving units was hospital care, including nursing/caring staff and premises costs. Coodacre & Calvert [29] recommend short periods of observation with exercise stress testing rather than overnight admission as a reasonably cost-effective treatment of patients with NCCP.

On average, 14%, 18%, and 25% of the patients with NCCP, AMI and AP were on sick-leave annually with a mean length of 103, 54 and 94 days respectively. These percentages were lower than those reported by Eslick & Talley [7], where 29% of patients with NCCP and 25% with cardiac chest pain were absent from work due to chest pain. The participants in the present study had substantially longer periods of absence from work compared to the 22 days reported by Eslick & Talley. We also found that about 10% of patients with NCCP received a disability pension, which was similar to those with AMI and AP. However, as reported in a previous study [15], there were some high users in this cohort who contributed to high indirect costs.

The annual societal cost of NCCP per patient was €10,068, which was lower than the costs of patients with AMI and AP, which were €15,989 and €14,737 respectively. To obtain a rough indication of the burden of these patient groups on society, the costs could be extrapolated to a larger context. In 2010, 81,121 patients (2.2% of all patients) were diagnosed with NCCP, 22,836 with AMI and 27,683 with AP in Sweden [30]. Due to the high prevalence of NCCP, the cumulative annual national cost of patients with NCCP would be about €817 million if all patients incurred the same costs as in the present study. The corresponding cost for patients with AMI and AP would then be about €364 million and €408 million respectively.

### Strengths and weaknesses

Our results are based on national databases; all healthcare and social insurance utilization over a 2-year period is reported. The study has also identified the most cost driving units in healthcare in connection with patients with NCCP, AMI and AP. This information is of great interest when developing new interventions for these patients in order to support them and reduce societal costs.

All costs associated with the patients’ healthcare utilization and productivity loss were included in the analysis. These also include costs that may have not been related to NCCP, AMI or AP. It should be acknowledged that this fact probably causes a slight over-estimation of the costs. However, including only the costs directly related to the diagnoses was not regarded to be feasible, since many symptoms are indirect consequences of the diagnoses that would then be excluded. Therefore, including only directly related costs would mean a marked under-estimation of the costs.

Data regarding sick-leave periods shorter than 15 days are not registered and are hence not included in the present analysis. Since short-term sick-leave data are unknown, this could mean that the indirect costs are under-estimated.

Not including costs related to an individual due to pain and suffering, or to family members for caring for the individual, or home care services could mean that the total societal costs for these patients are under-estimated.

## Conclusions and clinical implications

In general, patients with NCCP used significant amount of healthcare resources and were often absent from work. The annual societal cost of these patients, €10,068, was the lowest of the 3 groups. However, due to high prevalence of NCCP, the cumulative annual national costs of these patients could be more than the double of AMI and AP if all patients incurred the same costs as in this study. Further research is needed, including development and evaluation of psycho-educational treatment strategies to patients with NCCP, targeting management of chest pain and related symptoms, and thereby reducing healthcare utilization and costs**.**

## Competing interests

The authors declare that they have no competing interests to disclose.

## Authors’ contributions

All authors have been involved in the whole process writing this article, including design and/or analysis and interpretation of data, and drafting the manuscript. All authors have read and approved the final manuscript.

## Pre-publication history

The pre-publication history for this paper can be accessed here:

http://www.biomedcentral.com/1472-6963/13/403/prepub
